# A Study on the Correlations of Anxiety and Depression With Self-Management Ability and Endogenous Creatinine Clearance Rate in Renal Transplant Recipients

**DOI:** 10.3389/fpsyt.2021.715509

**Published:** 2021-09-28

**Authors:** ShiMin Hu, Yu Li, MingTao Quan, ShuJuan Yang, ZhaoMei Wan, XiaoYong Yan, ZhouKe Tan, GuoBiao Liang

**Affiliations:** ^1^Department of Nephropathy and Rheumatology, Affiliated Hospital of Zunyi Medical University, Zunyi, China; ^2^Nursing Department, Affiliated Hospital of Zunyi Medical University, Zunyi, China

**Keywords:** anxiety, depression, self-management, creatinine clearance rate, renal transplant recipients

## Abstract

**Objective:** To explore the effects of anxiety and depression on the self-management ability and endogenous creatinine clearance rate of renal transplant patients.

**Method:** Eighty-eight renal transplant recipients who were followed up in the outpatient clinic of the Affiliated Hospital of Zunyi Medical University were selected using convenient sampling. The self-made general data sheet, Self-Rating Anxiety Scale, Self-Rating Depression Scale, and Self-Management Scale for Kidney Transplant Recipients were used. Correlation analysis was used to find factors related to endogenous creatinine clearance, while multiple linear regression was used to identify factors influencing endogenous creatinine clearance. Patients with or without anxiety and depression were divided into groups, and the indexes of the groups were compared using the independent samples *t* test, rank-sum test, or chi-squared test.

**Results:** Anxiety was present in 12.5% of patients, depression in 25%, and a moderate level of self-management in 34.1%. Only 9.1% of renal transplant recipients had endogenous creatinine clearance within the normal range, and 34.1% had a body mass index not in the normal range (25% were overweight, and 9.1% were underweight). The endogenous creatinine clearance rate was negatively correlated with age and degree of depression, and positively correlated with body mass index, treatment management score, and psychosocial management score. The main influencing factors of endogenous creatinine clearance rate were age, sex, depression, body mass index, and treatment management score. The endogenous creatinine clearance rate and psychosocial management ability were significantly higher in patients without anxiety and depression than in patients with anxiety and depression (all *P* < 0.05).

**Conclusions:** Anxiety and depression showed significant negative effects on the psychosocial self-management ability and endogenous creatinine clearance rate of renal transplant recipients and thus should be given more attention.

## Introduction

Renal transplantation is currently recognized by the international medical community as the best treatment for patients with end-stage renal disease that can significantly improve survival and quality of life (1, 2). However, while patients benefit from the procedure, they also face many challenges, such as the need to make immunosuppressants for a long time and undergo regular monitoring, occurrence of rejection reactions, and risk of infection after transplantation (3). They also experience higher incidences of diabetes, tumor, and other comorbidities (4, 5). Therefore, a series of self-management programs are required after renal transplantation, and an important guarantee for long-term survival and good quality of life in kidney transplant patients is good self-management. Kidney transplant recipients are also prone to anxiety, depression and other negative emotions after surgery, which can affect the recovery and prognosis of patients (6). Arapaslan et al. (7) have shown that 50% of renal transplant recipients experience anxiety and 25% experience severe depression after surgery. However, the associations of anxiety and depression with self-management and renal function have not been well documented. Endogenous creatinine clearance is a crucial index to assess the damage of glomerular filtration function and evaluate renal function. At present, it has replaced the glomerular filtration rate as the standard for staging chronic renal insufficiency in clinical practice.

Thus, anxiety and depression can severely affect the recovery of renal function of renal transplant recipients, and adequate self-management is critical for renal survival and long-term quality of life of renal transplant recipients. To date, we have not found research on the relationship between anxiety, depression, self-management, and endogenous creatinine clearance in renal transplant recipients.

This study therefore aimed to explore the correlations of anxiety and depression with self-management and endogenous creatinine clearance in renal transplant recipients. Our findings could faciliate future interventions to better improve postoperative quality of life and achieve optimal health outcomes in these recipients.

## Materials and Methods

### Participants

Renal transplant recipients from the outpatient department of the Affiliated Hospital of Zunyi Medical University were selected using convenience sampling between June 2020 and December 2020. The inclusion criteria were as follows: patients who received their first allogeneic kidney transplant; age of ≥18 years; kidney transplantat time of ≥1 month; patients with communication and reading comprehension abilities. Patients with previous mental illness, patients who were taking antidepressants, patients who were experiencing other diseases affecting self-care, and patients with multiple organ transplants were excluded. All patients enrolled in this study volunteered to participate.

### Tools

**General information:** The general data sheet of kidney transplant recipients was designed by the researchers according to the purpose of the study and mainly included the patients' height, weight, marital status, educational level, occupation, economic status, payment method for treatment, and serum creatinine value.

**Anxiety level:** We used the Self-rating Anxiety scale (SAS) (8), which consists of 20 items scored in a scale of 1–4: 1 = no or little time, 2 = a small part of time, 3 = considerable time, and 4 = most or all of the time. The higher the SAS score, the higher the anxiety tendency. Based on this score, the degrees of anxiety were divided into three levels: mild anxiety (SAS score 50–59), moderate anxiety (SAS score 60–69), and severe anxiety (SAS score >69). The SAS has good reliability and validity, with Cronbach's alpha coefficients above 0.75.

**Depression level:** The Self-Rating Depression Scale (SDS) (9), has 20 items, and the scoring formula is the same as that of the SAS. The higher the score, the higher the tendency of depression. According to the score, the degree of depression was divided into three grades: 53–62 for mild depression, 63–72 for moderate depression, and >72 for severe depression. The SDS also has good reliability and validity, with Cronbach's alpha coefficients above 0.75.

**Self-management ability:** We used the Self-Management for renal transplant recipients (10), which consists of 28 items and the following four dimensions: diet management, treatment management, physical activity management. Each item adopts a four-level scoring system of 1–4, and the total score is 112. The higher the score, the better the self-management ability of the renal transplant recipients. A score of <68 indicated poor self-management ability, 68–90 medium self-management ability, and >90 good self-management ability. The scale has good reliability and validity, with a content validity index of 0.928 and a total Cronbach's alpha coefficient of 0.899. For each subscale, Cronbach's alpha coefficients range from 0.725 to 0.783 (all > 0.7), showing good internal consistency.

Endogenous creatinine clearance is calculated by the following: Endogenous creatinine clearance rate (CCR) = {[140 − age (years)] × body mass (kg)}/[0.818 × serum creatinine (Scr) (μ mol/L)], and the calculation result for women × 0.85. In adults, if the endogenous creatinine clearance rate is below 80 ml/min, the glomerular filtration function is decreased; reduced to 70~51 ml/min, mild damage; reduced to 50~31 ml/min, moderate damage; reduced to below 30 ml/minute, severe damage; reduced to 20~10 ml/min, early renal insufficiency; reduced to 10~5 ml/min, late renal insufficiency; and less than 5 ml/min, end-stage renal insufficiency. To obtain the BMI, the following equation is used: BMI = weight (kg)/(height × height) (m). According to the national standard for judging adult weight (National Health and Family Planning Commission of the people's Republic of China, 2013), BMI was divided into four groups: thin (BMI < 18.5), normal (18.5 ≤ BMI < 24), overweight (24 ≤ BMI < 28), and obese (BMI ≥ 28).

### Procedure

This study was approved by the Ethics Committee of the Affiliated Hospital of Zunyi Medical University (approval number, KLLY-2020-013). Trained renal transplantation follow-up nurses conducted an on-the-spot questionnaire survey of renal transplant recipients who met the inclusion criteria. The patients were informed about the purpose and content of this study in an anonymous, confidential, and voluntary manner. The questionnaire was distributed on the spot and recycled on the spot. A total of 88 questionnaires were sent out, and all of them were collected (effective recovery rate of 100%).

### Statistical Analysis

Count data were expressed as frequencies and percentages, and measurement data as means and standard deviations. Rank variables were analyzed with Spearman's correlation analysis, and continuous variables with Pearson's correlation analysis. We analyzed factors related to endogenous creatinine clearance using multiple linear regression to identify the factors influencing endogenous creatinine clearance. At the same time, differences in the endogenous creatinine clearance rate and self-management ability between the anxiety non-anxiety groups, and between depression and non-depression groups were compared. Measurement data with a normal distribution were compared using the independent samples *t*-tests, Measurement data with a normal distribution were expressed as M (P25, P75), and the rank-sum test was used for intergroup comparison. The classified variables were expressed by frequency and percentage, and the chi-squared test was used for comparison between groups. The difference was statistically significant when *P* < 0.05. All statistical analyses were performed using SPSS 18.0 (IBM Corp., Armonk, NY, USA).

## Results

A total of 88 participants were enrolled in this study. Table 1 reports the demographic characteristics of the participants, including their age, marital status, education, income, and mode of payment for treatment. The average age of the participants was 39.03 ± 11.00 years. The mean follow-up time was 23.70 ± 12.26 months.

**Table 1:** Patients' demographic characteristics (*n* = 88).

	**Number of patients**	**Proportion (%)**
Sex		
Male	58	65.9
Female	30	34.1
Marital status		
Unmarried	14	15.9
Married	71	80.7
Divorced	2	2.3
Widowed	1	1.1
Education		
Primary school	4	4.5
Middle school	25	28.4
High school	24	27.3
Bachelor's degree	35	39.8
Monthly income (yuan)		
0–2,000	36	40.9
2,001–4,999	19	21.6
5,000–7,999	25	28.4
≥8,000	8	9.1
Payment method		
Medical insurance	81	92
At one's own expense	4	4.5
Others	3	3.4

The SAS, SDS, and self-management ability scale scores of the renal transplant patients indicated the following: 11 (12.5%) participants experienced anxiety [10 (11.4%) with mild anxiety and 1 (1.1%) with moderate anxiety] and 22 (25%) experienced depression [16 (18.2%) with mild depression and 6 (6.8%) with moderate depression]. Furthermore, there were 30 (34.1%) participants with a medium level of self-management. The self-management scores for diet, treatment, physical activity, and social psychology were 3.36 ± 0.42, 3.56 ± 0.32, 3.31 ± 0.42, and 3.10 ± 0.49, respectively. The specific scores of each scale are shown in Table 2.

**Table 2:** Scores of the Self-Rating Anxiety Scale, Self-Rating Depression Scale, and Self-Management Scale for Kidney Transplant Recipients (*n* = 88).

**Scale**	**Minimum**	**Maximum**	**Total** **(***X ± s***)**
SAS	25	70	41.65 (mres)
SDS	32	67.5	47.42 (mres)
Self-Management Scale			
Diet	21	36	30.26
Treatment	26	40	35.64
Physical activity	10	20	16.58
Psychosocial	6	16	12.43
Total score	70	111	94.91

*SAS, Self-Rating Anxiety Scale; SDS, Self-Rating Depression Scale; Diet, diet management score; Treat, treatment management score; Physical activity, physical activity management score; Psychosocial, psychosocial management score*.

The average endogenous creatinine clearance rate of the included patients was 64.79 ± 22.55, among which, only 8 (9.1%) patients had normal creatinine clearance rate, 12 (13.6%) had CCR < 80 ml/min, 35 (39.8%) had CCR = 50–70 ml/min, 30 (34.1%) had CCR = 31–50 ml/min, and 3 (3.4%) had CCR < 30 ml/min. The average BMI was 21.96 ± 3.13, in which 58 (65.9%) patients had a body weight within the normal range. Among the patients, 22 (25%) were overweight, of whom 2 (2.2%) were obese. There were 8 (9.1%) patients who were underweight.

The results of the correlation analysis showed that there was no correlation between endogenous creatinine clearance and degree of anxiety in patients. Endogenous creatinine clearance was negatively correlated with age and degree of depression, and positively correlated with body mass index, treatment management score, and psychosocial management score. The point-two-column correlation coefficient with sex was −0.255 (Table 3).

**Table 3:** Analysis of the correlations of endogenous creatinine clearance and self-management with anxiety and depression.

	**CCR**	**Age**	**BMI**	**D**	**T**	**SP**	**PA**	**Total**	**Anxiety**	**Depression**	**Sex**
CCR	1										
Age	−0.337^**^	1									
BMI	0.260^*^	0.091	1								
D	−0.015	0.115	−0.132	1							
T	0.254^*^	−0.083	−0.081	0.648^**^	1						
PS	0.243^*^	−0.199	−0.096	0.343^**^	0.492^**^	1					
PA	0.084	−0.096	−0.071	0.750^**^	0.752^**^	0.482^**^	1				
Total	0.152	−0.046	−0.118	0.875^**^	0.885^**^	0.631^**^	0.895^**^	1			
Anxiety	−0.153	−0.021	−0.088	−0.088	−0.16	−0.384^**^	−0.228^*^	−0.224^*^	1		
Depression	−0.264^*^	0.003	−0.101	−0.053	−0.192	−0.395^**^	−0.152	−0.206	0.760^**^	1	
Sex	−0.255^*^	0.103	−0.18	0.032	−0.015	0.061	0.085	0.04	−0.205	−0.104	1

*CCR, creatinine clearance rate; BMI, body mass index; D, diet management score; T, treatment management score; PS, psychosocial management score; PA, physical activity management score; Total, total score of the self-management scale; Anxiety, patients with anxiety. Depression, patients with depression*.

* *P < 0.05*,

** *P < 0.01*.

Endogenous creatinine clearance was taken as the dependent variable, and six statistically significant variables (age, sex, depression, body mass index, treatment management score, psychosocial management score) in the correlation analysis and univariate analysis were taken as independent variables. The results of the multiple linear regression analysis showed that age, sex, depression, body mass index, and treatment management scores were the main influencing factors of endogenous creatinine clearance which accounted for 33.3% of the total variation (Table 4).

**Table 4:** Multiple linear regression of factors influencing endogenous creatinine clearance.

**Factor**	**B**	**SE**	**β**	**t**	**P**
(Constant)	39.407	34.895		1.129	0.262
Age	−0.66	0.188	−0.322	−3.516	0.001
BMI	1.782	0.673	0.248	2.649	0.01
Treatment management	1.397	0.64	0.203	2.183	0.032
Depression	−0.533	0.227	−0.219	−2.34	0.021
Sex	−9.345	4.412	−0.198	−2.118	0.037

*R = 0.577, R^2^ = 0.333. After adjustment: R^2^ = 0.292, F = 8.170, P < 0.001. SE, standard error*.

**Figure 1:**
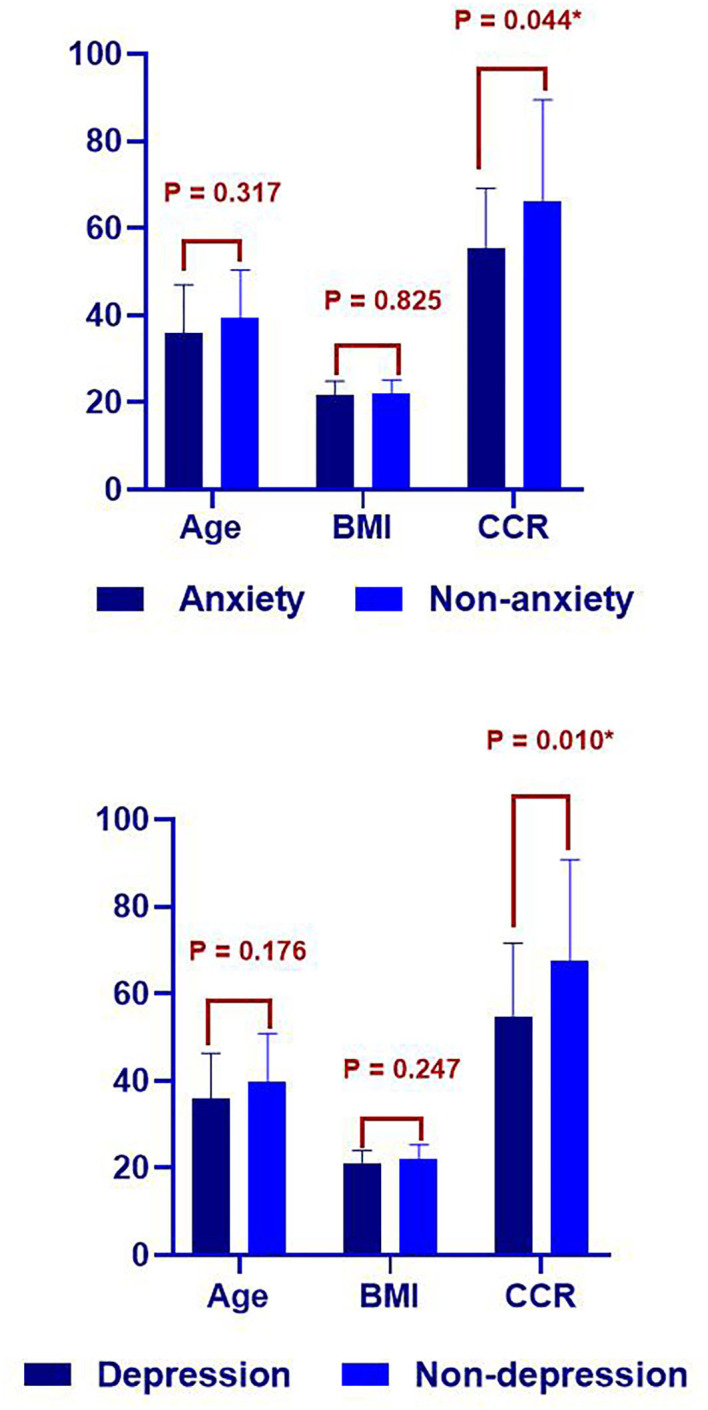
Comparison of age, body mass index (BMI), and creatinine clearance rate (CCR) between the anxiety and non-anxiety groups, and the depression and non-depression groups.

In the comparison of the self-management ability between patients with anxiety and depression and those without anxiety and depression, the age, body mass index, and endogenous creatinine clearance rate followed a normal distribution. Using the independent samples *t*-test, we found that the age and body mass index of patients with anxiety and depression were higher than those without anxiety and depression; however, the difference was not statistically significant (*P* > 0.05). The endogenous creatinine clearance rate in the anxiety and depression groups was significantly lower than that in the non-anxiety group and depression groups, and the difference was statistically significant (*P* < 0.05) (Figure 1). The total score of the self-management scale and the score of each dimension did not follow a normal distribution, which was expressed by M (P25, P75). When the rank-sum test was used to compare the groups, the results showed that the scores for diet management, treatment management, physical activity management, and psychosocial management and the total score of self-management scale in the anxiety and depression groups were lower than those in the non-anxiety and depression groups. However, only the dimension of psychosocial management showed a statistically significant difference *(P* < 0.05). Sex, payment method for treatment, educational level, and monthly income were expressed in terms of frequency and percentage. When the groups were compared using the chi-square test, the results showed that the proportion of patients with high school education and above in the non-anxiety group (71.3%) was significantly higher than that in the anxiety group (36.2%), and the proportion of patients with health insurance payments in the non-anxiety group (94.8%) was significantly higher than that in the anxiety group (72.7%). The proportion of patients with high school education and above in the non-depression group (75.7%) was significantly higher than that in the depression group (40.8%), and the difference was statistically significant (all *P* < 0.05) (Table 5).

**Table 5:** Comparison of creatine clearance, self-management ability, and general data between the anxiety and depression groups.

**Index**	**Anxiety groups**				**Depression groups**			
	**Anxiety**	**Non-anxiety**	**Z/X**	***P***	**Depression**	**Non-depression**	**Z/X**	***P***
	**(***n*** = 11)**	**(*n* = 77)**			**(***n*** = 19)**	**(***n*** = 69)**		
Sex (*n*, %)			1.14	0.23			0.65	0.41
Male	9 (81.8)	49 (63.6)			14 (73.7)	44 (63.8)		
Female	2 (18.2)	28 (36.4)			5 (26.3)	25 (36.2)		
DE (*n*, %)			−2.72	<0.01			−2.54	<0.05
Primary school or lower	2 (18.2)	2 (2.5)			2 (9.0)	2 (3.0)		
Junior middle school	5 (45.4)	20 (25.9)			11 (50)	14 (21.2)		
High school or technical secondary school	3 (27.2)	21 (27.2)			4 (18.1)	20 (30.3)		
Junior college or bachelor's degree	1 (9.0)	34 (44.1)			5 (22.7)	30 (45.4)		
Monthly income (*n*, %, yuan)			−2.72	0.06			−1.52	0.12
0–2,000	8 (72.7)	28 (36.3)			13 (59.0)	23 (31.8)		
2,001–4,999	0 (0)	19 (26.4)			2 (9.0)	17 (25.7)		
5,000–7,999	3 (27.2)	22 (28.5)			6 (27.2)	19 (28.70		
≥8,000	0 (0)	8 (10.3)			1 (4.5)	7 (10.6)		
Payment method			6.84	<0.05			2.31	0.31
Medical insurance	8 (72.7)	73 (94.8)			16 (84.2)	65 (94.2)		
At one's own expense	2 (18.2)	2 (2.6)			2 (10.5)	2 (2.9)		
Others	1 (9.1)	2 (2.6)			1 (5.3)	2 (2.9)		
D	28.5 (26.2, 34.5)	30 (28, 34)	−1.11	0.26	30 (26, 34)	30 (28, 34)	−0.48	0.62
T	36 (30.5, 39)	37 (34, 38)	−0.97	0.33	36 (32, 38)	37 (34, 38)	−0.44	0.65
SP	15.5 (13.2, 18.5)	17 (15, 18)	−1.87	0.06	16 (14, 19)	17 (15, 18)	−0.9	0.36
PA	11 (10, 13.2)	12 (11, 14)	−2.7	<0.01	11 (10, 12)	12 (11, 14)	−2.96	<0.01
Total	86.5 (81, 105.7)	98 (88.5, 102)	−1.7	0.08	92.47 ± 11.59	95.59 ± 8.74	−1.1	0.27

*DE, degree of education; D, diet management score; T, treatment management score; PS, psychosocial management score; PA, physical activity management score; Total, total score of the self-management scales*.

## Discussion

Studies have confirmed that after organ transplantation, 20–60% of recipients experience anxiety, depression, or psychosocial pain (11). In our study, the percentages of patients with anxiety and depression were 12.5% and 25%, respectively, which are higher than those reported by Czyzewski et al. (11.3 and 11.9%, respectively) (12) and lower than those reported by Gök et al. (84 and 66%, respectively) (13, 14).

Our research showed that both anxiety and depression could lead to a significant decrease in the patients' self-management ability, especially in the psychosocial aspect. In our study, 34.1% of the renal transplant recipients had a medium level of self-management, and among the four dimensions of self management, psychosocial management scored the lowest. This finding is consistent with the results of Xie et al. (15). Psychological factors and negative emotions are well-known risk factors of poor quality of life in renal transplant recipients (16). Anxiety can affect the patients' quality of life by influencing their psychological management (11), while depression can lead to a twofold increase in the risk of transplant failure and death (17). As reported previously, self-management plays an important role in preventing the development of chronic diseases, reducing the incidence of complications, and improving the quality of life (18). Moreover, adhering to a good self-management program is an important factor related to graft survival and medium- and long-term quality of life in renal transplant recipients. However, the correlations of anxiety and depression with patients' self-management ability and CCR have not been well documented.

In this study, we analyzed the general data of patients and the relationships of anxiety and depression with self-management ability and endogenous creatinine clearance. We found that endogenous creatinine clearance was negatively correlated with age and degree of depression and positively correlated with body mass index, treatment management score and psychosocial management score, but it showed no correlation with the degree of anxiety of the patient. In other words, the higher the degree of depression, the lower the endogenous creatinine clearance rate, but the higher the level of self-management in treatment and psychosocial aspects, the higher the endogenous creatinine clearance rate.

We further analyzed the factors affecting the endogenous creatinine clearance rate and found that sex, age, body mass index, treatment management ability, and depression were the main influencing factors of endogenous creatinine clearance, which could explain 33.3% of the total variation. Although age and sex are factors that we cannot modify, we can guide patients to maintain a good body mass index through reasonable diet and exercise, and improve their physical function (19).

Aside from the same influencing factors found in our and previous studies, self-management ability and depression were also independent influencing factors of endogenous creatinine clearance found in this study. The self-management of treatment among renal transplant recipients mainly includes taking drugs according to doctor's advice, getting used to the effects and side effects of the drugs, self-monitoring body temperature, blood pressure, and urine volume, and paying regular revisit to the docyor (20). The level of treatment management ability has a significant impact on the endogenous creatinine clearance rate of patients. Thus, medical staff should pay attention to the cultivation of patients' treatment management ability. It is also very necessary to screen patients for depression to provide a basis for targeted treatment and nursing measures.

Creatinine clearance is the most important index for evaluating renal function. To our knowledge, this study is the first to analyze the correlations of anxiety and depression with self-management and creatinine clearance in renal transplant recipients. We compared the general data, endogenous creatinine clearance rate, and self-management ability of patients with or without anxiety and depression, and found that patients with a higher educational level and health insurance payment support had a lower incidence of anxiety and depression. Patients without anxiety or depression had a higher psychosocial management ability and higher endogenous creatinine clearance. These findings suggests that for patients with a lower level of education, we should adopt health education and communication methods that are easier and more acceptable to them, and strengthen their knowledge and understanding of renal transplantation and self-management. As mentioned by Schmid-Mohler et al. (21), health insurance support would also be necessary for transplant patients. Once again, our results confirmed the significant effect of depression on endogenous creatinine clearance. Thus, it is important to screen renal transplant recipients for anxiety and depression to promote targeted interventions, especially psychosocial interventions. We also found that the incidence of depression was higher than expected; therefore, the management of depression could be considerably significant in improving the CCR of patients. This implies that treatment and nursing interventions alone may not be adequate to solve these psychosocial problems of these patients. A psychological consultation team with knowledge of kidney transplantation is needed (22).

Our study has some limitations. We only investigated renal transplant recipients in one hospital, and the sample size was limited. This setup may not provide a complete picture of the correlations of anxiety and depression with self-management and creatinine clearance in renal transplant recipients. Another limitation is that because *t*-tests were used, potential confounding of age and gender on the association of pyschological factors and clearance cannot be determined. Nevertheless, the findings of this study have an important practical significance because they showed the negative effects of anxiety and depression on self-management, and the negative effects of depression on creatinine clearance. Therefore, we should screen renal transplant recipients for anxiety and depression and comprehensively evaluate factors such as the patient's age, cultural background, socioeconomic status, and family relationships before surgery. A psychological service team with a professional knowledge of transplantation should be established to provide more-comprehensive health management services for transplant patients.

## Data Availability Statement

The raw data supporting the conclusions of this article will be made available by the authors, without undue reservation.

## Ethics Statement

The studies involving human participants were reviewed and approved by Ethics Committee of Affiliated Hospital of Zunyi Medical University. The patients/participants provided their written informed consent to participate in this study. Written informed consent was obtained from the individual(s) for the publication of any potentially identifiable images or data included in this article.

## Author Contributions

All authors listed have made a substantial, direct and intellectual contribution to the work, and approved it for publication.

## Funding

The Education Department Fund Project of Guizhou Province, Grant No. KY (2017) 045.

## Conflict of Interest

The authors declare that the research was conducted in the absence of any commercial or financial relationships that could be construed as a potential conflict of interest.

## Publisher's Note

All claims expressed in this article are solely those of the authors and do not necessarily represent those of their affiliated organizations, or those of the publisher, the editors and the reviewers. Any product that may be evaluated in this article, or claim that may be made by its manufacturer, is not guaranteed or endorsed by the publisher.

## Abbreviations

CCR: creatinine clearance rate
SAS: Self-Rating Anxiety Scale
SDS: Self-rating Depression Scale.
